# Association between preconception serum lipid levels and pregnancy complications in singleton live births from IVF/ICSI with fresh embryo transfers

**DOI:** 10.3389/fnut.2026.1915181

**Published:** 2026-10-21

**Authors:** Wang-Yu Cai, Xi Luo, Qing Zhang, Hongping Li, Huadi Yang, Xinyan Wang, Jian Xu

**Affiliations:** 1 Department of Gynecology, First Affiliated Hospital, Zhejiang Chinese Medical University, Hangzhou, China; 2 Department of Gynecology, Second Affiliated Hospital, Zhejiang Chinese Medical University, Hangzhou, China; 3 Department of Gynecology, Zhejiang Hospital, Hangzhou, China; 4 Department of Gynecology, Affiliated Hangzhou First People's Hospital, Westlake University School of Medicine, Hangzhou, China; 5 Fourth Affiliated Hospital, Zhejiang University School of Medicine, Yiwu, China; 6 Women’s Hospital, Zhejiang University School of Medicine, Hangzhou, China

**Keywords:** assisted reproduction, gestational diabetes mellitus, preconception lipid, pregnancy complication, preterm delivery

## Abstract

**Purpose:**

This study aimed to examine how preconception serum lipid profiles are associated with pregnancy complications in women who achieved singleton live births following assisted reproductive technology (ART).

**Methods:**

This study included 1,036 women who had singleton births after ART. Preconception serum lipid measurements included low-density lipoprotein cholesterol (LDL-C), high-density lipoprotein cholesterol (HDL-C), total cholesterol (TC), and triglycerides (TG). The pregnancy complication outcomes included cesarean section, gestational diabetes mellitus (GDM), preeclampsia, preterm delivery (PTD), small for gestational age (SGA), large for gestational age (LGA), low birthweight (LBW), macrosomia, and neonatal intensive care unit (NICU) admission.

**Results:**

The incidence of cesarean section, GDM, preeclampsia, PTD, SGA, LGA, LBW, macrosomia, and NICU admission was 51.6%, 3.8%, 0.7%, 7.6%, 6.8%, 15.6%, 6.4%, 5.7%, and 1.8%, respectively. Women with dyslipidemia had higher rates of GDM (11.7% vs. 2.1%, *p* < 0.001), PTD (13.9% vs. 6.3%, *p* < 0.001), and LWB (10.6% vs. 5.5%, *p* = 0.012). After adjusting for possible confounders, TG (OR 1.49[1.11–1.99]) was positively associated with PTD. TC (OR 1.48[1.08–2.02]), LDL-C (OR 1.48[1.03–2.14]), and HDL-C (OR 3.03[1.19–7.73]) were positively associated with SGA. TC (OR 2.22[1.63–3.04]) and TG (OR 1.20[1.00–1.44]) were positively associated with GDM.

**Conclusion:**

Preconception serum lipid levels were associated with pregnancy complications in women receiving ART.

## Introduction

The number of children conceived through assisted reproductive technology (ART) has increased in recent years, and evidence suggests that there are adverse pregnancy and obstetric outcomes associated with singleton, twin, and multiple pregnancies resulting from ART (1–3). Although various studies have attempted to explain the underlying reasons for this higher risk, no clear agreement has been reached. It appears that this higher risk may be linked to factors intrinsic to the infertile couple, such as dysfunction in steroidogenesis, oocyte quality, endometrial function, and placental function. Another explanation is that the ART procedure may not fully simulate the conditions of a natural pregnancy.

Therefore, it is urgent to identify factors associated with pregnancy complication outcomes following ART. Some studies have reported predictors of adverse pregnancy outcomes in women undergoing ART. For example, unpromising endometrial receptivity might be one reason. After fresh cycles for singleton pregnancies after ART, small for gestational age (SGA) infants were more frequent in women with thinner endometrial thickness (4). Thin endometrial thickness was also related to preterm delivery (PTD) and low birthweight (LBW) in frozen cycles for singleton pregnancies after ART (5). Furthermore, endocrine factors might be another cause. Elevated estradiol levels during the trigger phase are considered independent predictors of LBW and SGA (6). In addition, PTD and LBW risks were increased when hormone replacement therapy was employed for endometrial preparation during frozen blastocyst transfers (7).

With the rising prevalence of obesity worldwide (8), lipid abnormalities have become very common (9, 10). For lipid health evaluation, low-density lipoprotein cholesterol (LDL-C), high-density lipoprotein cholesterol (HDL-C), total cholesterol (TC), and triglycerides (TG) are the most commonly measured indicators. Significant associations have been established between serum lipid levels and female reproductive health, including endometrial receptivity, oocyte development, and placental function. Studies have shown that substantial quantities of lipids, both within cells and in the extracellular space, accumulate in the decidua basalis and frequently extend into the superficial myometrium (11). In pregnancies affected by preeclampsia, the placental spiral arteries exhibit pathological changes, notably the presence of foam cells filled with lipids (12). The majority of studies reported the relationships between pregnancy lipid levels and pregnancy outcomes. For example, elevated first-trimester TG levels may be the key risk factor for adverse pregnancy outcomes in the general obstetric population (13). Recent evidence indicates that elevated preconception serum lipid levels correlate with reduced live birth rates and increased miscarriage risk after ART (14, 15). A study also reported that both low and high prepregnancy cholesterol levels were related to higher PTD risk in natural pregnancies (16). However, no studies reported the association between preconception serum lipid levels and pregnancy complication outcomes following ART.

We examined how preconception serum lipid profiles relate to pregnancy complications in women who achieved singleton live births through ART, including cesarean section, gestational diabetes mellitus (GDM), preeclampsia, PTD, SGA, large for gestational age (LGA), LBW, macrosomia, and neonatal intensive care unit (NICU) admission.

## Materials and methods

### Study design

The current study protocol was approved by the ethics committee of a university-based hospital. The study was conducted in accordance with the Declaration of Helsinki. All women received ART treatment and delivered a singleton live birth in the hospital from January 2019 to January 2021. This was a retrospective study and did not include any patient information; therefore, the ethics committee waived the requirement for informed consent.

### Study population

This study included women aged 20–45 years who achieved a singleton live birth after ART from January 2019 to January 2021. All women were required to have a fresh embryo transfer through either *in vitro* fertilization (IVF) or intracytoplasmic sperm injection (ICSI) cycle. All women were required to have a confirmed single gestational sac on ultrasound at 5–7 weeks after the embryo transfer, while cases involving vanishing twin syndrome were not included. Live birth was defined as the delivery of a viable infant at gestational age greater than or equal to 28 weeks. All causes of infertility were included. The inclusion criteria were as follows: (1) singleton live birth, (2) embryo transfer, (3) IVF or ICSI cycle, and (4) available preconception serum lipid data. Women were excluded if they had (1) multiple pregnancies; (2) unavailable serum lipid data; (3) unavailable pregnancy complication outcome data; and (4) missing important relevant data. A total of 1,036 women were included.

Before ART treatment, demographic data including age, height, weight, and current smoking status were recorded. Body mass index (BMI) was calculated based on height and weight. Infertility-related data included the infertility factor, whether it was primary or secondary infertility, and the duration of infertility. Sex hormones included anti-Mullerian hormone (AMH), basal follicle-stimulating hormone (FSH), and basal estradiol (E2). Metabolic hormones included LDL-C, HDL-C, TC, TG, and glucose. Dyslipidemia was diagnosed based on any one of the following criteria: HDL-C < 1.0 mmol/L; LDL-C ≥ 4.1 mmol/L; TC ≥ 6.2 mmol/L; or TG ≥ 2.3 mmol/L. These criteria have been validated in the Chinese population (17).

### Preconception serum lipid panel

All blood samples were acquired while fasting during days 2–4 of the menstrual cycle. Metabolic hormone concentrations were quantified using a Beckman AU5800 chemistry analyzer (Beckman Coulter, USA). Glucose levels were measured using the hexokinase glucose-6-phosphate dehydrogenase method. TC was measured using the cholesterol oxidase enzymatic method, LDL-C and HDL-C were analyzed using the direct enzymatic method, and TG was analyzed using the GPO-PAP method. Sex hormones were measured by a ROCHE E801 analyzer using an electrochemiluminescence immunoassay method. Intra-assay coefficients of variation were <1.53%, and inter-assay coefficients of variation were <3.55% for all hormones.

### Outcome measures

The pregnancy complication outcomes included cesarean section, GDM, preeclampsia, PTD, SGA, LGA, LBW, macrosomia, and NICU admission. PTD was defined as a delivery that occurred at a gestational age of <37 weeks. GDM and preeclampsia were screened for among all women receiving obstetric care at the same hospital. GDM was screened between 24 and 28 weeks of gestation and was diagnosed if any plasma glucose value met or exceeded the following thresholds: 5.1 mmol/L when fasting, 10.0 mmol/L at 1 h, or 8.5 mmol/L at 2 h. Preeclampsia was screened every month before 28 gestational weeks, every 2 weeks before 36 gestational weeks, and every week after 36 gestational weeks. Preeclampsia was diagnosed based on the occurrence of combined gestational hypertension (defined as diastolic blood pressure >90 mmHg and/or systolic pressure >140 mmHg) and proteinuria after 20 weeks of gestation. The diagnosis of LGA (defined as >90th percentile) or SGA (defined as <10th percentile) was based on the standards set by the Chinese government (18). Neonates were classified by birth weight as LBW (<2,500 g), normal (2,500–4,000 g), or macrosomia (>4,000 g). NICU admission was recorded in the hospital registry.

### Statistical analysis

SPSS Statistics 24.0 (IBM) was used to perform data analyses. All baseline characteristics of women were compared between women with dyslipidemia and those without dyslipidemia. Continuous variables were compared using independent samples *t*-tests, and categorical variables were compared using chi-squared tests. Pregnancy complication outcomes were compared using chi-square tests in women with and without dyslipidemia.

As this is a retrospective study, the associations between serum lipid levels and pregnancy complications were exploratory. A univariate logistic regression analysis was performed to examine the associations between each serum lipid level and pregnancy complication outcomes separately, and the results were reported as odds ratios (ORs) with 95% confidence intervals (95% CIs). Second, adjusted ORs and 95% CIs were calculated by multivariate logistic regression analyses, after adjusting for possible confounders. Age, BMI, and glucose levels were important clinical indicators associated with pregnancy outcomes; therefore, they were identified as confounders and adjusted for in subsequent analyses. For variables with possible skewness, Spearman’s correlation analysis was performed, and the correlation coefficient ρ was reported. All *p*-values were two-tailed, with a *p*-value of < 0.05 considered statistically significant.

## Results

### Characteristics of study population

A total of 1,530 live births were recorded between January 2019 and January 2021. The reasons for exclusion were as follows: multiple pregnancies (*n* = 311), unknown serum lipid levels (*n* = 101), and missing infant information (*n* = 82; Figure 1). Table 1 presents the baseline data of all included women. The average age of the women was 30.8 ± 3.6 years, and they had an average BMI of 21.7 ± 2.8 kg/m^2^. Reproductive characteristics, sex hormones, metabolic hormones, and ovarian stimulation outcomes are reported in Table 1.

**Figure 1:**
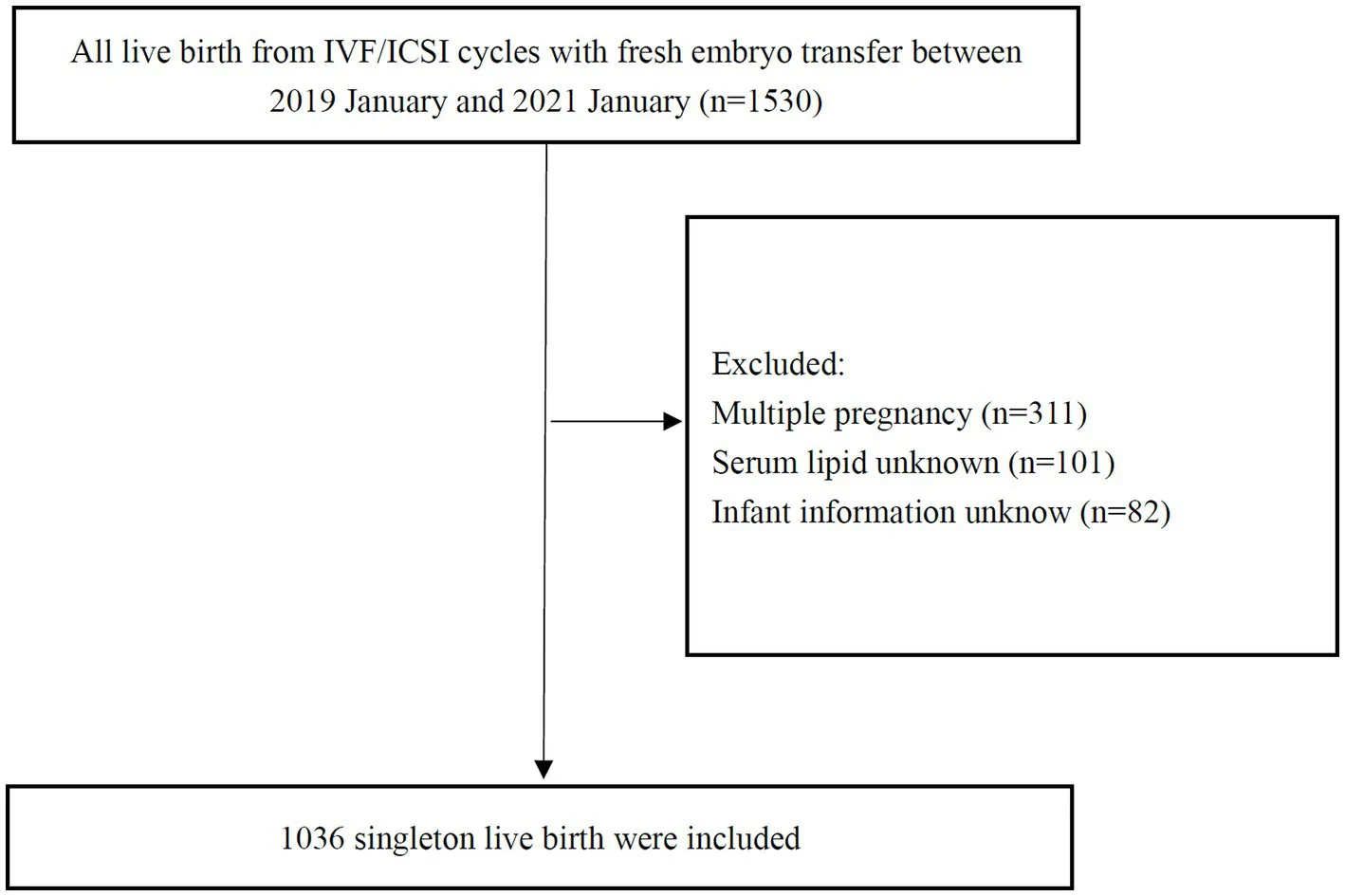
Flowchart for selecting women included in this study.

**Table 1:** Characteristics of included women.

Variables	*N*	Mean ± SD or frequency
Age (year)	1,036	30.8 ± 3.6
BMI (kg/m^2^)	1,036	21.7 ± 2.8
Infertility diagnosis		
Tubal factor	405	39.1
Diminished ovarian reserve	89	8.6
Male	156	15.1
Ovulation disorder	44	4.2
Endometriosis	56	5.4
Unexplained	92	8.9
Mixed	153	14.8
Other	41	4.0
Current smoking	8	0.8%
Duration of infertility (years)	1,036	3.2 ± 2.6
Primary infertility	511	49.3
AMH (ng/ml)	973	3.3 ± 2.3
Basal FSH (IU/l)	1,036	6.7 ± 2.8
Basal E2 (pmol/l)	1,036	146.5 ± 382.7
Glucose (mmol/l)	952	5.1 ± 0.5
Total cholesterol (mmol/l)	1,036	4.4 ± 0.8
HDL-cholesterol (mmol/l)	1,036	1.3 ± 0.3
Triglycerides (mmol/l)	1,036	1.1 ± 1.0
LDL-cholesterol (mmol/l)	1,036	2.6 ± 0.7
Total gonadotropin (IU)	1,036	2,222.3 ± 772.7
Days of stimulation	1,036	10.2 ± 2.3
Peak E2 (pmol/l)	1,003	9,045.4 ± 4,435.2
Oocytes retrieved	1,036	9.5 ± 4.2
ART method		
IVF	734	70.8
ICSI	302	29.2
Number of embryos transferred	1,036	1.8 ± 0.4
Endometrial thickness	1,020	10.7 ± 2.3

Continuous data are presented as mean ± standard deviation.

BMI: body mass index, AMH: anti-Mullerian hormone, FSH: follicle-stimulating hormone, HDL: high-density lipoprotein, LDL: low-density lipoprotein, ART: assisted reproductive technology, IVF: in vitro fertilization, ICSI: intracytoplasmic sperm injection.

Table 2 compared baseline data between women with and without dyslipidemia. BMI was higher in women with dyslipidemia (23.2 ± 3.0 vs. 21.4 ± 2.6). The frequency of infertility diagnosis was statistically different. Basal FSH levels were lower in women with dyslipidemia (5.8 ± 2.4 vs. 6.8 ± 2.8). Women with dyslipidemia had higher levels of glucose (5.2 ± 0.5 vs. 5.1 ± 0.5), TC (4.9 ± 1.3 vs. 4.3 ± 0.7), LDL-C (3.0 ± 1.0 vs. 2.5 ± 0.6), and TG (2.1 ± 2.0 vs. 1.0 ± 0.4), while they had lower levels of HDL-C (1.1 ± 0.3 vs. 1.4 ± 0.3) levels. The endometrial thickness on the day of triggering was higher in women with dyslipidemia (11.1 ± 2.3 vs. 10.6 ± 2.3).

**Table 2:** Characteristics of included women with and without dyslipidemia.

Variables	Not dyslipidemia (*n* = 856)	Dyslipidemia (*n* = 180)	*p* value
Age (year)	30.7 ± 3.6	31.3 ± 3.6	0.053
BMI (kg/m^2^)	21.4 ± 2.6	23.2 ± 3.0	<0.001
Infertility diagnosis			0.008
Tubal factor	337 (39.4)	68 (37.8)	
Diminished ovarian reserve	73 (8.5)	16 (8.9)	
Male	126 (14.7)	30 (16.7)	
Ovulation disorder	27 (3.2)	17 (9.4)	
Endometriosis	45 (5.3)	11 (6.1)	
Unexplained	83 (9.7)	9 (5.0)	
Mixed	131 (15.3)	22 (12.2)	
Other	34 (4.0)	7 (3.9)	
Current smoking	6 (0.7)	2 (1.1)	0.568
Duration of infertility (year)	3.1 ± 2.5	3.4 ± 2.8	0.225
Primary infertility	421 (49.2)	90 (50.0)	0.648
AMH (ng/ml)	3.3 ± 2.2	3.5 ± 2.7	0.265
Basal FSH (IU/l)	6.8 ± 2.8	5.8 ± 2.4	<0.001
Basal E2 (pmol/l)	152.5 ± 418.7	118.0 ± 93.3	0.272
Glucose (mmol/l)	5.1 ± 0.5	5.2 ± 0.5	<0.001
Total cholesterol (mmol/l)	4.3 ± 0.7	4.9 ± 1.3	<0.001
HDL-cholesterol (mmol/l)	1.4 ± 0.3	1.1 ± 0.3	<0.001
Triglycerides (mmol/l)	1.0 ± 0.4	2.1 ± 2.0	<0.001
LDL-cholesterol (mmol/l)	2.5 ± 0.6	3.0 ± 1.0	<0.001
Total gonadotropin (IU)	2,207.2 ± 753.7	2,293.9 ± 856.0	0.171
Days of stimulation	10.1 ± 2.1	10.5 ± 2.8	0.086
Peak E2 (pmol/l)	9,206.0 ± 4,484.4	8,290.6 ± 4,125.6	0.013
Oocytes retrieved	9.5 ± 4.1	9.5 ± 4.4	0.852
ART method			0.524
IVF	610 (71.3)	124 (68.9)	
ICIS	246 (28.7)	56 (31.1)	
Number of embryos transferred	1.8 ± 0.4	1.8 ± 0.4	0.144
Endometrial thickness	10.6 ± 2.3	11.1 ± 2.3	0.017

BMI: body mass index, AMH: anti-Mullerian hormone, FSH: follicle-stimulating hormone, HDL: high-density lipoprotein, LDL: low-density lipoprotein, ART: assisted reproductive technology, IVF: in vitro fertilization, ICSI: intracytoplasmic sperm injection.

### Pregnancy complication outcomes

In our study, the incidence of C-section, GDM, preeclampsia, PTD, SGA, LGA, LBW, macrosomia, and NICU admission was 51.6%, 3.8%, 0.7%, 7.6%, 6.8%, 15.6%, 6.4%, 5.7%, and 1.8%, respectively. Table 3 presents the pregnancy complication outcomes and compared them between the dyslipidemia group and the normal serum lipid group. Dyslipidemia was associated with higher rates of GDM (11.7% vs. 2.1%), PTD (13.9% vs. 6.3%), and LWB (10.6% vs. 5.5%).

**Table 3:** Pregnancy complication outcomes in women with and without dyslipidemia.

Variables	Total (*n* = 1,036)	Not dyslipidemia (*n* = 856)	Dyslipidemia (*n* = 180)	*p* value
PTD	79 (7.6)	54 (6.3)	25 (13.9)	**<0.001**
Cesarean section	535 (51.6)	435 (50.8)	100 (55.6)	0.248
GDM	39 (3.8)	18 (2.1)	21 (11.7)	**<0.001**
Preeclampsia	7 (0.7)	4 (0.5)	3 (1.7)	0.074
SGA	71 (6.8)	63 (7.4)	8 (4.4)	0.156
LGA	162 (15.6)	134 (15.7)	28 (15.6)	0.959
LBW	66 (6.4)	47 (5.5)	19 (10.6)	**0.012**
Macrosomia	59 (5.7)	47 (5.5)	12 (6.7)	0.543
NICU	19 (1.8)	13 (1.5)	6 (3.3)	0.099

GDM: gestational diabetes mellitus, PTD: preterm delivery, SGA: small for gestational age, LGA: large for gestational age, LBW: low birthweight, NICU: neonatal intensive care unit. Bold values indicates statistical significances.

Table 4 reported the association between preconception serum lipid levels and pregnancy complication outcomes before adjusting for confounders. Higher TG (OR per one unit = 1.47 [1.12–1.93]) levels were positively associated with PTD. Higher levels of TC (OR per one unit = 1.51 [1.12–2.04]), LDL-C (OR per one unit = 1.45 [1.01–2.07]), and HDL-C (OR per one unit = 2.98 [1.229–7.29]) were positively associated with SGA. Lower levels of HDL-C (OR per one unit = 0.51 [0.28–0.94]) was associated with LGA. Higher levels of TC (OR per one unit = 2.40 [1.78–3.23]) and TG (OR per one unit = 1.23 [1.01–1.51]) were positively associated with GDM. Lower levels of HDL-C (OR per one unit = 0.64 [0.42–0.98]) was associated with cesarean section. Due to the low incidence of preeclampsia, we compared lipid levels between women with and without the condition; TC (4.8 ± 1.3 vs. 4.4 ± 0.8, *p* = 0.290), TG (1.4 ± 0.7 vs. 1.2 ± 1.0, *p* = 0.548), LDL-C (2.6 ± 0.9 vs. 2.6 ± 0.7, *p* = 0.963), and HDL-C (1.3 ± 0.2 vs. 1.3 ± 0.3, *p* = 0.431) were all comparable. To address possible skewness in TG values, Spearman’s correlation analysis was performed, and TG levels were associated with PTD (*ρ* = 0.219, *p* < 0.01), LBW (*ρ* = 0.123, *p* < 0.01), GDM (*ρ* = 0.198, *p* < 0.01), NICU admission (*ρ* = 0.075, *p* = 0.015), and cesarean section (*ρ* = 0.086, *p* = 0.006). However, TG was not associated with SGA (*ρ* = −0.035, *p* = 0.261), LGA (*ρ* = 0.058, *p* = 0.06), macrosomia (*ρ* = 0.024, *p* = 0.450), and preeclampsia (*ρ* = 0.031, *p* = 0.321).

**Table 4:** Unadjusted association between serum lipids and pregnancy complication outcomes.

Outcomes	TC		TG		LDL		HDL	
	OR (95% CI)	*p* value	OR (95% CI)	*p* value	OR (95% CI)	*p* value	OR (95% CI)	*p* value
Cesarean section	0.96 (0.83–1.11)	0.563	1.15 (0.96–1.36)	0.123	0.98 (0.83–1.16)	0.815	**0.64 (0.42–0.98)**	**0.041**
GDM	**2.40 (1.78–3.23)**	**<0.001**	**1.23 (1.01–1.51)**	**0.041**	1.45 (0.99–2.14)	0.058	1.09 (0.36–3.29)	0.875
Preeclampsia	1.49 (0.71–3.11)	0.288	1.10 (0.79–1.53)	0.577	0.98 (0.35–2.76)	0.963	0.31 (0.02–5.65)	0.426
PTD	1.13 (0.87–1.47)	0.344	**1.47 (1.12–1.93)**	**0.006**	1.19 (0.88–1.61)	0.260	0.89 (0.39–1.99)	0.768
SGA	**1.51 (1.12–2.04)**	**0.007**	0.99 (0.73–1.35)	0.950	**1.45 (1.01–2.07)**	**0.042**	**2.98 (1.22–7.29)**	**0.017**
LGA	0.97 (0.80–1.17)	0.721	1.16 (0.97–1.38)	0.098	0.91 (0.72–1.14)	0.390	**0.51 (0.28–0.94)**	**0.030**
LBW	1.24 (0.94–1.63)	0.125	1.19 (1.00–1.42)	0.055	1.18 (0.85–1.64)	0.324	1.56 (0.68–3.56)	0.290
Macrosomia	0.94 (0.69–1.29)	0.716	1.21 (1.00–1.46)	0.051	0.95 (0.66–1.38)	0.800	0.42 (0.15–1.13)	0.086
NICU	1.25 (0.76–2.04)	0.380	1.13 (0.94–1.36)	0.211	1.49 (0.88–2.53)	0.141	0.54 (0.10–2.95)	0.480

GDM: gestational diabetes mellitus, PTD: preterm delivery, SGA: small for gestational age, LGA: large for gestational age, LBW: low birthweight, NICU: neonatal intensive care unit. Bold values indicates statistical significances.

Table 5 presents the association between preconception serum lipid levels and pregnancy complication outcomes, after adjusting for confounders. A total of 952 women with complete data were included in the analyses. In multivariable analyses, the incidence of C-section, GDM, preeclampsia, PTD, SGA, LGA, LBW, macrosomia, and NICU admission was 486 (51.6%), 38 (4.0%), 7 (0.7%), 75 (7.9%), 68 (7.1%), 147 (15.4%), 64 (6.7%), 54 (5.7%), and 18 (1.9%), respectively. Higher levels of TG (OR per one unit = 1.49 [1.11–1.99]) were positively associated with PTD. Higher levels of TC (OR per one unit = 1.48 [1.08–2.02]), LDL-C (OR per one unit = 1.48 [1.02–2.14]), and HDL-C (OR per one unit = 3.03 [1.19–7.73]) were positively associated with SGA. Higher levels of TC (OR per one unit = 2.36 [1.74–3.21]) and TG (OR per one unit = 1.21 [1.00–1.46]) were positively associated with GDM.

**Table 5:** Adjusted association between serum lipids and pregnancy complication outcomes.

Outcomes	TC		TG		LDL		HDL	
	OR (95% CI)	*p* value	OR (95% CI)	*p* value	OR (95% CI)	*p* value	OR (95% CI)	*p* value
PTD	1.18 (0.90–1.53)	0.225	**1.49 (1.11–1.99)**	**0.009**	1.20 (0.88–1.64)	0.250	1.17 (0.50–2.72)	0.717
Cesarean section	0.92 (0.79–1.07)	0.259	1.09 (0.92–1.29)	0.342	0.92 (0.77–1.10)	0.372	0.72 (0.45–1.15)	0.172
GDM	**2.36 (1.74–3.21)**	**<0.001**	**1.21 (1.00–1.46)**	**0.048**	1.41 (0.94–2.11)	0.099	1.28 (0.40–4.09)	0.678
Preeclampsia	1.42 (0.67–3.01)	0.365	1.07 (0.75–1.54)	0.711	0.84 (0.29–2.46)	0.753	0.43 (0.02–8.88)	0.588
SGA	**1.48 (1.08–2.02)**	**0.014**	0.98 (0.70–1.34)	0.895	**1.48 (1.02–2.14)**	**0.039**	**3.03 (1.19–7.73)**	**0.020**
LGA	0.91 (0.75–1.10)	0.327	1.06 (0.92–1.22)	0.448	0.82 (0.64–1.04)	0.102	0.76 (0.41–1.45)	0.424
LBW	1.26 (0.95–1.67)	0.106	1.22 (1.00–1.50)	0.053	1.21 (0.86–1.69)	0.271	1.56 (0.65–3.74)	0.318
Macrosomia	0.88 (0.63–1.23)	0.451	1.16 (0.97–1.38)	0.103	0.81 (0.54–1.22)	0.318	0.67 (0.23–1.95)	0.460
NICU	1.11 (0.67–1.86)	0.684	1.08 (0.88–1.32)	0.454	1.30 (0.73–2.32)	0.365	0.81 (0.12–4.74)	0.817

Adjusted for age, BMI, and glucose.

GDM: gestational diabetes mellitus, PTD: preterm delivery, SGA: small for gestational age, LGA: large for gestational age, LBW: low birthweight, NICU: neonatal intensive care unit. Bold values indicates statistical significances.

## Discussion

Our study results showed that elevated preconception serum lipid levels were associated with an increased risk of pregnancy complications, including PTD, SGA, and GDM, in singleton live births following ART. Furthermore, these associations still existed after adjusting for potential confounders such as preconception BMI and glucose levels. These results suggested the importance of lipid screening and management before assisted reproduction treatment.

Our results suggested that increasing TC and TG levels were positively associated with GDM; increased TG levels were positively associated with PTD; and increased TC, LDL-C, and HDL-C levels were positively associated with SGA. Published research has focused on the associations between neonatal outcomes and maternal lipid levels during different trimesters of pregnancy. In rural Gambia, rising lipid concentrations during pregnancy correlated with greater infant birth weight and reduced risks of LBW and SGA (19). Among Chinese women, elevated TG levels in the third trimester independently increased the risk of macrosomia while lowering the likelihood of SGA (20). Elevated maternal TG and TC levels in both early and late pregnancy independently predicted GDM in otherwise healthy pregnant women (20, 21). A retrospective study involving 12,715 women in a maternity center who had singleton births and underwent routine serum lipid screening in both early (9–13 weeks) and late (28–42 weeks) pregnancy found that persistently high TG levels throughout pregnancy further increased the risk of GDM (13). Another retrospective study of 49,612 women with singleton pregnancies in a maternity center found that high TG levels during early pregnancy increased the risk of PTD not only in obese or overweight women but also in women with normal BMI (22).

Limited articles reported the effect of preconception lipid levels on pregnancy complications. In a multicenter, longitudinal, observational study designed to describe the development of risk factors for coronary heart disease in young Black and white men and women, both high and low prepregnancy cholesterol levels were associated with the risk of PTD in women with at least one singleton birth during 20 years of follow-up (16). Our results first reported that increasing preconception serum lipid levels are associated with higher risks of SGA and LBW in singleton live births after ART. Adverse pregnancy outcomes, such as GDM and PTD, are the most common pregnancy complications and cause a great burden in obstetric settings, especially in ART pregnancies; therefore, clinical interventions aimed at managing preconception serum lipid levels may be beneficial in reducing pregnancy complications after assisted reproduction treatment.

Preconception lipid levels are closely related to lipid levels during pregnancy; therefore, their mechanisms on pregnancy outcomes partly overlap. Normally, lipid levels gradually increase with the progress of pregnancy, and this increase sustains the demand of physiological changes and fetal growth. Increasing preconception lipid levels represent a proatherogenic phenotype among women with ART and might influence glucose homeostasis and endothelial function during pregnancy. Hypertriglyceridemia at the start of pregnancy is closely related to impaired insulin action and β-cell function (23). Abnormal lipid metabolism also reflects unpromising endothelial function, which may compromise placental function (24). Placental dysfunction, which is usually considered in pregnancies conceived after ART, is a possible explanation of some pregnancy complications including early miscarriage, pregnancy-related hypertension, stillbirth, and preterm delivery (25–29). Fetal growth restriction is also associated with endothelial dysfunction. Impaired vascular remodeling and decreased vascular volume affect placental vascularity, resulting in decreased oxygen supply to the fetus and reduced growth of the fetus, which are associated with PTD and SGA.

Furthermore, preconception serum lipid levels may directly affect female reproductive health before pregnancy, since serum lipids influence steroidogenesis, folliculogenesis, and embryo quality. In the female reproductive system, crucial hormones including progesterone, estrogens, androgens, and glucocorticoids are all synthesized from lipids through steroidogenesis (30). *De novo* lipogenesis is rare in the ovaries; therefore, steroidogenesis in the ovaries depends on lipids absorbed from the body’s circulation. In the ovarian microenvironment, cholesterol mainly originates from HDL-C (31). Peak E2 levels on the day of the trigger injection predict ART pregnancy outcomes (6). In vitrified-warmed cycles involving single embryo transfer, poor embryo quality would result in a significantly higher risk of LBW (32). Dysregulated preconception serum lipids may impair ovarian steroidogenesis and oocyte quality, ultimately influencing female fecundity.

In addition, endometrial receptivity is another reason. Commonly used measures to evaluate endometrial receptivity include endometrial thickness and certain protein markers. After a fresh IVF/ICSI cycle, the risk of SGA was increased twofold in women with thin endometrial thickness (4). A thinner endometrial thickness was also an independent predictor for a higher likelihood of PTD for singletons born after fresh embryo transfer (33). A previous study found that high progesterone levels during controlled ovarian hyperstimulation are associated with dysregulated lipid metabolism in the endometrium during the peri-implantation period, which may impair endometrial receptivity and early embryo implantation (34).

Interestingly, HDL-C was positively, rather than negatively, associated with the risk of SGA. The transport of cholesterol from the maternal to the fetal circulation by the placenta mainly depends on LDL uptake through the LDL receptor, while HDL transports excess cholesterol to the liver for metabolism (35). Therefore, higher HDL levels may reduce cholesterol uptake in the placenta, resulting in an insufficient supply to the fetus.

The rising global prevalence of dyslipidemia has heightened concerns about its impact on female reproductive health. Our findings provide a possible biological basis for a shared mechanistic pathway linking reduced female reproduction and cardiovascular conditions. The results of the current study suggest that preconception serum lipid levels are associated with the risk of pregnancy complications in women undergoing assisted reproduction treatment. Unfavorable preconception lipid profiles can be improved through lifestyle changes, dietary adjustments, or statin therapy (36–38). Our results further emphasize the importance of lipid factors in female reproductive health.

It is reasonable to hypothesize that treatment to improve preconception serum lipid levels may decrease the risk of pregnancy complications in assisted reproduction. Prospective clinical trials focus on the associations between interventions such as lifestyle modification and pregnancy complications, since they have no obvious negative effect on female reproductive function.

The major strength of the current study is that it is a robust statistical analysis with a large sample size, which investigated the associations between preconception serum lipid levels and pregnancy complications, after adjusting for main potential confounders. However, several limitations should also be noted. First, only women who achieved singleton live births after fresh embryo transfers in a single-center cohort of homogeneous ethnicity were included, which was a selection bias and would reduce the generalizability of the study results. Second, the included cohort had a relatively low incidence of GDM and preeclampsia, younger age and lower BMI are plausible reasons for this finding. Additionally, unrecorded diagnoses in other medical settings was another possible reason. Both aspects may introduce selection bias. Third, the retrospective design did not include a suitable sample size calculation, and multiple comparisons increased the rate of false positives. For rare outcomes such as preeclampsia and NICU admission, it was difficult to acquire robust associations between lipid levels and these outcomes. Future research with a larger sample size is necessary. In addition, confounding bias could not be excluded completely due to the retrospective design of the study. Finally, because women with different infertility factors were included, future research should investigate the effect of preconception serum lipid levels on pregnancy complications in specific cohorts, such as patients with polyendocrine metabolic ovarian syndrome patients.

## Conclusion

This study demonstrates that pre-pregnancy serum lipid levels are associated with pregnancy complications following assisted reproduction technology. For women preparing for assisted reproduction treatment, lipid screening may be beneficial to evaluate the risk of pregnancy complications. Future research could investigate whether improving lipid levels before assisted reproduction treatment is associated with pregnancy complications.

## Data Availability

The original contributions presented in the study are included in the article/supplementary material, further inquiries can be directed to the corresponding author.

## References

[1] Qin J Liu X Sheng X Wang H Gao S. Assisted reproductive technology and the risk of pregnancy-related complications and adverse pregnancy outcomes in singleton pregnancies: a meta-analysis of cohort studies. Fertil Steril. (2016) 105:73–85.e6. doi: 10.1016/j.fertnstert.2015.09.007, 26453266

[2] Qin J Wang H Sheng X Liang D Tan H Xia J. Pregnancy-related complications and adverse pregnancy outcomes in multiple pregnancies resulting from assisted reproductive technology: a meta-analysis of cohort studies. Fertil Steril. (2015) 103:1492–1508.e7. doi: 10.1016/j.fertnstert.2015.03.018, 25910567

[3] Qin JB Wang H Sheng X Xie Q Gao S. Assisted reproductive technology and risk of adverse obstetric outcomes in dichorionic twin pregnancies: a systematic review and meta-analysis. Fertil Steril. (2016) 105:1180–92. doi: 10.1016/j.fertnstert.2015.12.131, 26801066

[4] Guo Z Xu X Zhang L Zhang L Yan L Ma J. Endometrial thickness is associated with incidence of small-for-gestational-age infants in fresh in vitro fertilization-intracytoplasmic sperm injection and embryo transfer cycles. Fertil Steril. (2020) 113:745–52. doi: 10.1016/j.fertnstert.2019.12.014, 32147172

[5] Hu K-L Kawai A Hunt S Li W Li X Zhang R . Endometrial thickness in the prediction of neonatal adverse outcomes in frozen cycles for singleton pregnancies. Reprod Biomed Online. (2021) 43:553–60. doi: 10.1016/j.rbmo.2021.04.014, 34332902

[6] Royster GD Krishnamoorthy K Csokmay JM Yauger BJ Chason RJ DeCherney AH . Are intracytoplasmic sperm injection and high serum estradiol compounding risk factors for adverse obstetric outcomes in assisted reproductive technology? Fertil Steril. (2016) 106:363–370.e3. doi: 10.1016/j.fertnstert.2016.04.023, 27172401 PMC4975645

[7] Zong L Liu P Zhou L Wei D Ding L Qin Y. Increased risk of maternal and neonatal complications in hormone replacement therapy cycles in frozen embryo transfer. Reprod Biol Endocrinol. (2020) 18:36. doi: 10.1186/s12958-020-00601-3, 32366332 PMC7199365

[8] NCD Risk Factor Collaboration (NCD-RisC). Worldwide trends in body-mass index, underweight, overweight, and obesity from 1975 to 2016: a pooled analysis of 2416 population-based measurement studies in 128·9 million children, adolescents, and adults. Lancet. (2017) 390:2627–42. doi: 10.1016/S0140-6736(17)32129-3, 29029897 PMC5735219

[9] Tóth PP Potter D Ming EE. Prevalence of lipid abnormalities in the United States: the National Health and Nutrition Examination Survey 2003–2006. J Clin Lipidol. (2012) 6:325–30. doi: 10.1016/j.jacl.2012.05.002, 22836069

[10] Huang Y Gao L Xie X Tan SC. Epidemiology of dyslipidemia in Chinese adults: meta-analysis of prevalence, awareness, treatment, and control. Popul Health Metrics. (2014) 12:28. doi: 10.1186/s12963-014-0028-7, 25371655 PMC4219092

[11] Brosens I Brosens JJ Muter J Benagiano G. Acute atherosis and diffuse lipid infiltration of the placental bed: a review of historical lipid studies. Placenta. (2020) 97:36–41. doi: 10.1016/j.placenta.2020.06.012, 32792060

[12] Stevens DU Al-Nasiry S Bulten J Spaanderman MEA. Decidual vasculopathy and adverse perinatal outcome in preeclamptic pregnancy. Placenta. (2012) 33:630–3. doi: 10.1016/j.placenta.2012.04.013, 22656691

[13] Xue R-H Wu D-D Zhou C-L Chen L Li J Li Z-Z . Association of high maternal triglyceride levels early and late in pregnancy with adverse outcomes: a retrospective cohort study. J Clin Lipidol. (2021) 15:162–72. doi: 10.1016/j.jacl.2020.10.001, 33144084

[14] Cai W-Y Luo X Chen E Lv H Fu K Wu X-K . Serum lipid levels and treatment outcomes in women undergoing assisted reproduction: a retrospective cohort study. Front Endocrinol (Lausanne). (2021) 12:633766. doi: 10.3389/fendo.2021.633766, 33763032 PMC7982671

[15] Yang T Zhao J Zhang Q Liu D Liu N Li Y . Associations between dyslipidaemia and pregnancy outcomes in the first complete cycle of IVF/ICSI: a real-world analysis. Reprod Biomed Online. (2021) 43:1095–105. doi: 10.1016/j.rbmo.2021.08.030, 34764017

[16] Catov JM Ness RB Wellons MF Jacobs DR Roberts JM Gunderson EP. Prepregnancy lipids related to preterm birth risk: the coronary artery risk development in young adults study. J Clin Endocrinol Metab. (2010) 95:3711–8. doi: 10.1210/jc.2009-2028, 20501685 PMC2913035

[17] Joint committee issued Chinese guideline for the management of dyslipidemia in adults. 2016 Chinese guideline for the management of dyslipidemia in adults. Zhonghua Xin Xue Guan Bing Za Zhi. (2016) 44:833–53. 10.3760/cma.j.issn.0253-3758.2016.10.005 27903370

[18] National Health Commission of the People’s Republic of China. Growth standard for newborns by gestational age. People’s Republic of China Health Industry Standard.(2022). Available online at: https://www.nhc.gov.cn/wjw/c100311/202208/07787ef64ba34fe1bc8bbdae9fd0d4e5.shtml

[19] Okala SG Sise EA Sosseh F Prentice AM Woollett LA Moore SE. Maternal plasma lipid levels across pregnancy and the risks of small-for-gestational age and low birth weight: a cohort study from rural Gambia. BMC Pregnancy Childbirth. (2020) 20:153. doi: 10.1186/s12884-020-2834-1, 32164563 PMC7068879

[20] Jin W-Y Lin S-L Hou R-L Chen X-Y Han T Jin Y . Associations between maternal lipid profile and pregnancy complications and perinatal outcomes: a population-based study from China. BMC Pregnancy Childbirth. (2016) 16:60. doi: 10.1186/s12884-016-0852-9, 27000102 PMC4802610

[21] Wang C Zhu W Wei Y Su R Feng H Hadar E . The associations between early pregnancy lipid profiles and pregnancy outcomes. J Perinatol. (2017) 37:127–33. doi: 10.1038/jp.2016.191, 27787507

[22] Lin X-H Wu D-D Li C Xu Y-J Gao L Lass G . Maternal high triglyceride levels during early pregnancy and risk of preterm delivery: a retrospective cohort study. J Clin Endocrinol Metab. (2019) 104:1249–58. doi: 10.1210/jc.2018-01372, 30321365

[23] Eppel D Feichtinger M Lindner T Kotzaeridi G Rosicky I Yerlikaya-Schatten G . Association between maternal triglycerides and disturbed glucose metabolism in pregnancy. Acta Diabetol. (2021) 58:459–65. doi: 10.1007/s00592-020-01644-z, 33387029 PMC8053660

[24] McElwain CJ Tuboly E McCarthy FP McCarthy CM. Mechanisms of endothelial dysfunction in pre-eclampsia and gestational diabetes mellitus: windows into future cardiometabolic health? Front Endocrinol (Lausanne). (2020) 11:655. doi: 10.3389/fendo.2020.00655, 33042016 PMC7516342

[25] Gun BD Numanoglu G Ozdamar SO. The comparison of vessels in elective and spontaneous abortion decidua in first trimester pregnancies: importance of vascular changes in early pregnancy losses. Acta Obstet Gynecol Scand. (2006) 85:402–6. doi: 10.1080/00016340500501731, 16612700

[26] Lambropoulou M Tamiolakis D Venizelos J Liberis V Galazios G Tsikouras P . Imbalance of mononuclear cell infiltrates in the placental tissue from foetuses after spontaneous abortion versus therapeutic termination from 8th to 12th weeks of gestational age. Clin Exp Med. (2006) 6:171–6. doi: 10.1007/s10238-006-0111-x, 17191109

[27] Guzin K Tomruk S Tuncay YA Naki M Sezginsoy S Zemheri E . The relation of increased uterine artery blood flow resistance and impaired trophoblast invasion in pre-eclamptic pregnancies. Arch Gynecol Obstet. (2005) 272:283–8. doi: 10.1007/s00404-005-0005-2, 16007505

[28] Romero R Kusanovic JP Chaiworapongsa T Hassan SS. Placental bed disorders in preterm labor, preterm PROM, spontaneous abortion and abruptio placentae. Best Pract Res Clin Obstet Gynaecol. (2011) 25:313–27. doi: 10.1016/j.bpobgyn.2011.02.006, 21388889 PMC3092823

[29] Wijaya JC Khanabdali R Georgiou HM Kokkinos MI James PF Brennecke SP . Functional changes in decidual mesenchymal stem/stromal cells are associated with spontaneous onset of labour. Mol Hum Reprod. (2020) 26:636–51. doi: 10.1093/molehr/gaaa045, 32609359

[30] Morel Y Roucher F Plotton I Goursaud C Tardy V Mallet D. Evolution of steroids during pregnancy: maternal, placental and fetal synthesis. Ann Endocrinol (Paris). (2016) 77:82–9. doi: 10.1016/j.ando.2016.04.023, 27155772

[31] Azhar S Tsai L Medicherla S Chandrasekher Y Giudice L Reaven E. Human granulosa cells use high density lipoprotein cholesterol for steroidogenesis. J Clin Endocrinol Metab. (1998) 83:983–91. doi: 10.1210/jcem.83.3.4662, 9506760

[32] Huang J Tao Y Zhang J Yang X Wu J Kuang Y . Poor embryo quality is associated with a higher risk of low birthweight in vitrified-warmed single embryo transfer cycles. Front Physiol. (2020) 11:415. doi: 10.3389/fphys.2020.00415, 32499716 PMC7243353

[33] Wu J Huang J Dong J Xiao X Li M Wang X. The thicker the endometrium, the better the neonatal outcomes? Hum Reprod Open. (2023) 2023:hoad028. doi: 10.1093/hropen/hoad028, 37489142 PMC10363027

[34] Li J Gao Y Guan L Zhang H Chen P Gong X . Lipid profiling of peri-implantation endometrium in patients with premature progesterone rise in the late follicular phase. J Clin Endocrinol Metabol. (2019) 104:5555–65. doi: 10.1210/jc.2019-00793, 31390011

[35] Ouimet M Barrett TJ Fisher EA. HDL and reverse cholesterol transport. Circ Res. (2019) 124:1505–18. doi: 10.1161/CIRCRESAHA.119.312617, 31071007 PMC6813799

[36] Hasan B Nayfeh T Alzuabi M Wang Z Kuchkuntla AR Prokop LJ . Weight loss and serum lipids in overweight and obese adults: a systematic review and meta-analysis. J Clin Endocrinol Metab. (2020) 105:3695–703. doi: 10.1210/clinem/dgaa673, 32954416

[37] Ge L Sadeghirad B Ball GDC da Costa BR Hitchcock CL Svendrovski A . Comparison of dietary macronutrient patterns of 14 popular named dietary programmes for weight and cardiovascular risk factor reduction in adults: systematic review and network meta-analysis of randomised trials. BMJ. (2020) 369:m696. doi: 10.1136/bmj.m696, 32238384 PMC7190064

[38] Delahoy PJ Magliano DJ Webb K Grobler M Liew D. The relationship between reduction in low-density lipoprotein cholesterol by statins and reduction in risk of cardiovascular outcomes: an updated meta-analysis. Clin Ther. (2009) 31:236–44. doi: 10.1016/j.clinthera.2009.02.017, 19302897

